# The Effect of Different Recovery Duration on Repeated Anaerobic Performance in Elite Cyclists

**DOI:** 10.1515/hukin-2015-0119

**Published:** 2015-12-30

**Authors:** Sultan Harbili

**Affiliations:** 1 Selçuk University, School of Physical Education and Sports, Alaeddin Keykubat Campus, 42003, Konya, Turkey

**Keywords:** repeated Wingate test, recovery, anaerobic power

## Abstract

This study investigated the effect of recovery duration on repeated anaerobic performance in elite cyclists. The study followed a cross-over design protocol. Twelve elite male cyclists were randomly assigned to three groups (with recovery duration of 1, 2 and 3 min, respectively). All the subjects performed 4 repeated Wingate tests (4 × 30 s WT) at 48 h intervals for three different recovery periods. No significant interaction was observed between the effects of recovery duration and repetition (p>0.05), whereas there was a significant main effect of repetition on peak power, mean power, and a fatigue index (p<0.05). Peak power decreased significantly in repeated WTs with 1 and with 2 min recovery duration (p<0.05), but it did not change significantly in a repeated WT with 3 min recovery (p>0.05). In contrast, mean power decreased significantly in repeated WTs with 1, 2 and 3 min recovery duration (p<0.05). The fatigue index increased significantly in a repeated WT with 1 min recovery duration (p<0.05), but no significant difference was observed in the fatigue index in repeated WTs with 2 and 3 min recovery (p>0.05). In a 4 × 30 s WT, peak power decreased in cycles with 1 and 2 min recovery duration, but remained unchanged with 3 min recovery duration, whereas mean power decreased in all recovery duration procedures. The WT with 1 min recovery duration caused greater fatigue. Although recovery duration affected both peak power and mean power, the effect on peak power was greater.

## Introduction

Shorter recovery duration plays a decisive role in short-term high-intensity exercise in elite athletes. The nature and duration of intervening recovery periods determine the ability to produce and maintain high power output during successive sprints required in many sport events (Glaister, 2005; Glaister et al., 2005). Intermittent high-intensity sprint efforts combined with short recovery periods have been identified as a key factor in physical performance (Jones and Cooper, 2014). Thus, numerous studies have focused on effects of a warm-up on anaerobic performance (Poprzęcki et al., 2007), as well as fatigue and recovery processes in repeated-sprint performance (Bogdanis et al., 1996; McGawley and Bishop, 2006; Dupont et al., 2007; Kohler et al., 2010). These studies have demonstrated a decrease in repeated-sprint performance closely related to the number of repetitions, exercise duration and the subject’s ability to recover from the periods of work.

Power output during the first 30 s in a maximal intensity exercise requires the ability to rapidly resynthesize adenosine triphosphate (ATP) from anaerobic sources, i.e. phosphocreatine (PCr) degradation and glycolytic pathways, as well as aerobic pathways, approximately 28, 56 and 16%, respectively (Hultman and Sjöholm, 1983; Smith and Hill, 1991; Faria et al., 2005). After a maximal intensity exercise, oxygen uptake is increased during recovery due to the replenishment of tissue oxygen stores, PCr resynthesis, the removal of lactate and inorganic phosphate (Bogdanis et al., 1995; Glaister et al., 2005). At the same time, lactate removal from the blood following exercise appears to be of great importance in improving the subsequent performance, particularly when the exercise is repeated at high intensity (Ahmaidi et al., 1996). Recovery duration plays a decisive role in the successful completion of these processes. Although a complete recovery affects each of these variables, the recovery of maximum power production is primarily dependent on PCr resynthesis (Bogdanis et al., 1995). Previous studies have found that the halftime of PCr resynthesis was ~22 s after maximal dynamic exercise (Harris et al., 1976); that after 30 s maximal sprint cycling, it took 1.5 min to replenish 65% of PCr stores (Bogdanis et al., 1995); and that the full return of PCr stores to resting levels took >3 min (Wragg et al., 2000; Spencer et al., 2006). Furthermore, Sahlin et al. (1979) reported that resynthesis of ATP to ~80–100% of resting levels required 2–4 min of recovery.

Repeated sprint protocols have typically involved a bout of 2 to 25 sprints of 4–10 s (Wragg et al., 2000; Bishop et al., 2001; Spencer et al., 2006; Kohler et al., 2010). The influence of recovery duration on repeated-sprint performance has been investigated using a range of methods and recovery periods of different duration (from 2–3 s to 120 s), with 30 s recovery being the most common (Bishop et al., 2001; Glaister et al., 2005; Billaut and Basset, 2007). In those studies, repeated-sprint performances were of shorter duration, involved multiple repetitions and shorter recovery periods, as is characteristic of team sports. In contrast, relatively few studies have investigated the effect of 30 s-4 min rest periods on recovery in sprint performances with fewer repetitions and longer duration (20–30 s) (Bogdanis et al., 1996; Dupont et al., 2007). However, those studies focused more on different recovery types (active and passive) than on recovery duration. In a recent study, Brown and Glaister (2014) reported that when rest periods were short, a passive recovery strategy appeared to optimize repeated short sprint (RSS) performance, and as the recovery duration increased, subsequent RSS performance appeared to benefit from an active recovery strategy. The effect of longer recovery duration of 6–9–12 min in 3 × 45 s repeated-sprint performances has only been investigated by one previous study (Ainswort et al., 1993).

The time ratio between recovery and exercise is a key factor in repeated-sprint performance. Thus, the present study was designed to examine the effects of various recovery duration on repeated anaerobic performance. For this purpose, the effect of recovery duration of 1, 2 and 3 min between maximal intensity exercise during a 30 s Wingate test (WT) on peak power (PP), mean power (MP), and fatigue index (FI) variables was investigated.

## Material and Methods

### Experimental Design

This study followed a cross-over design protocol. The 12 subjects included in the study performed 4 repeated-sprint cycle tests (Wingate protocol) at 48 hour intervals on 3 different days. The subjects were randomly assigned to three groups of four on the first test day; on each test day, while one group performed the test with 1 min recovery duration, another followed the same protocol with 2 min recovery duration, while the third group with 3 min rest periods. To avoid learning and fatigue effects, all the subjects had performed four repeated WTs at 48-hour intervals, each with different recovery duration, i.e., 1, 2 and 3 min. The subjects rested passively on the cycle ergometer seat between WTs. All WTs were performed between 3:00 pm and 6:00 pm. The subjects did not perform any other exercise 24 h pre-WTs and during the five-day WTs. All participants were instructed to avoid alcohol consumption and ensure adequate hydration 24 h before each trial and to refrain from consuming food and fluids (except water) for 2 h before each WT. The subjects ingested their daily food and fluid intake during the 24 hours before the first WT.

### Participants

Twelve elite male cyclists, who were national (n=10) and Olympic team (n=2, participants in the 2012 Summer Olympics in London) athletes with a minimal training load of 15 h per week during the season participated in this study. The mean age, body height, body mass, and training experience of participants were 21.50 ± 3.09 years, 1.78 ± 0.03 m, 67.94 ± 4.23 kg and 7.25 ± 2.42 years, respectively. This study was conducted in accordance with the guidelines set forth by the Institutional Review Board of the Selçuk University. After all subjects were fully informed about the risks and stresses associated with the research protocol, written informed consent was obtained from all subjects prior to study participation.

### Measures

#### Wingate Test

The subjects first warmed up on a cycle ergometer (Monark 894E, Stockholm, Sweden) for 5 min at a resistance of 50 W and at 90 rpm pre WT. After 3 min of rest, all subjects performed four 30 s WTs with 75 g·kg^−1^ body weight load with 1, 2 and 3 min recovery periods. The participants were verbally encouraged to maintain as high a pedaling rate as possible throughout the 30 s test duration. The Wingate test results were transferred to a computer via Monark Anaerobic Test Software 2.0. Peak power and mean power were calculated by this software. The fatigue index (FI) was calculated according to the following formula (Bar-Or, 1987): FI = (maximal pedal speed − minimal pedal speed) / maximal pedal speed * 100.

#### Heart rate (HR)

HR values of all the subjects were instantaneously recorded after 1 min rest in the sitting position in bed after the cyclists woke up at 9:00 in the morning, after 3 min in the sitting position on a chair prior to the warm up for the WT, immediately after WTs, and at the end of recovery periods of 1, 2 and 3 min (S610i, Polar Electro Oy, Kempele, Finland).

### Statistical analysis

The data were expressed as the mean ± standard deviation (SD). All variables measured were analyzed using a 3 (recovery duration (D): 1, 2 and 3 min) × 4 (repetition (R): WT1, WT2, WT3, and WT4) repeated measures analysis of variance (ANOVA). When a significant interaction or a main effect of factor 1 (recovery duration) or factor 2 (repetition) was observed, a one-way repeated measures analysis of variance with a Bonferroni post-hoc test was performed for multiple comparisons. The data were analyzed using SPSS 18 for Windows. The level of significance was set at 0.05.

## Results

No significant interactions (recovery duration × repetition) or main effect (recovery duration) were observed for PP, MP, and FI in different recovery duration (Tables 1, 2 and 3).

Repeated measures ANOVA results showed significant differences for PP in both 1 min recovery (F(3,33)=13.78, p<0.05) and 2 min recovery (F(3,33)=10.28, p<0.05) periods (Table 1). Furthermore, pairwise analyses confirmed that PP decreased significantly in repeated WTs with 1 and 2 min recovery durations (p<0.05). In contrast, the unchanged PP values found in a repeated WT with 3 min recovery duration confirmed that PP was sustained during the WTs (Table 1).

Significant differences were found for MP in repeated WTs with 1 (F(3,33)=43.03, p<0.05), 2 (F(3,33)=39.48, p<0.05) and 3 min recovery periods (F(3,33)=13.29, p<0.05). Pairwise analyses showed significant decreases for MP during WT2, WT3, and WT4 compared with WT1 in all recovery periods (Table 2).

Another significant difference was observed in the FI for 1 min recovery period (F(3,33)=8.13, p<0.05). Only the FI was significantly higher in WT4 than in WT1, WT2, and WT3 with 1 min recovery duration (Table 3).

The mean resting HR recorded when cyclists woke up was 52.0 ±3.1 b·min-1, and HRs pre WTs with 1, 2 and 3 min recovery periods were 59.8 ±3.6 b·min-1, 59.6 ±3.3 b·min-1, and 57.0 ±3.9 b·min-1, respectively.

**Figure 1:**
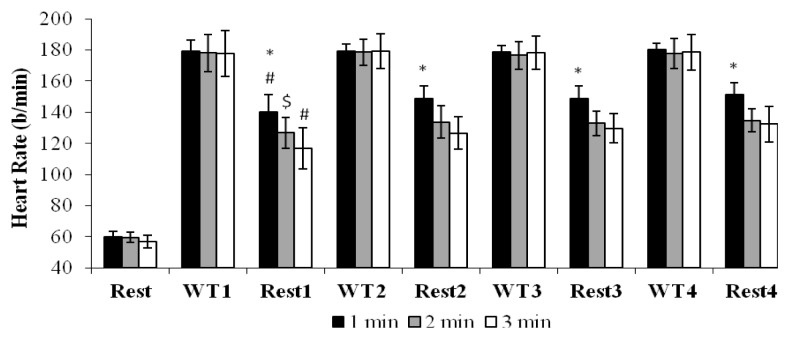
*, HR at the end of 1 min recovery duration was significantly higher than that of 2 min and 3 min recovery duration for all rest intervals (p<0.05 ). #, HR at the end of Rest1 for 1 and 3 min recovery duration was significantly lower than that of Rest2-Rest3-Rest4 (p<0.05). $, HR was significantly lower in Rest1 than in Rest4 in 2 min recovery duration (p<0.05).

No significant differences were observed in the HR obtained immediately after repeated WTs (p>0.05). When comparisons were made between groups, the HR at the end of 1 min recovery period was significantly higher than these of 2 and 3 min recovery periods for all rest intervals (F(2,33)=17.02, p<0.05). With regard to within-groups comparisons, in 1 and 3 min recovery periods, the HR at the end of Rest1 was significantly lower than that of Rest2, Rest3, or Rest4 (F(3,99)=27.77, p<0.05). In addition, the HR in Rest4 was significantly higher than in Rest1 for the 2 min recovery period (p<0.05) (Figure 1).

## Discussion

The most important finding of this study was that recovery duration had a greater effect on peak power, although it also affected mean power. Peak power decreased in repeated WTs with 1 and 2 min recovery periods, whereas no significant decrease was found for peak power in a repeated WT with a 3 min rest interval. The effect of recovery duration on repeated anaerobic performance had frequently been examined in studies with shorter recovery duration between relatively shorter multiple sprints. It was reported in a study of 20 × 5 s repeated sprints with 10 and 30 s recovery duration that 30 s recovery duration produced higher PP values (Dupont et al., 2007). It was also demonstrated that increased (from 10 to 50 s, with 5 s intervals) and decreased (from 50 to 10 s) recovery duration and constant recovery duration between 10 × 6 s cycling sprints were effective on PP, and that increasing recovery duration resulted in a smaller decrease in PP values (Billaut and Basset, 2007). In the current literature, maximal power output is associated primarily with resynthesis of PCr (Bogdanis et al., 1995); in addition, the half-time for PCr repletion was reported to be ~22 s or more (Harris et al., 1976), while full recovery of PCr stores required > 3 min (Meyer, 1988; Bogdanis et al., 1998). Therefore, as the recovery duration between repetitions increased, the subsequent sprint intensity became greater due to the recovery of PCr stores. In addition, in a study on 2 × 8 s sprints with 15, 30, 60 and 120 s recovery duration, PP decreased with 15 s recovery in both sexes, whereas it remained unchanged in other recovery duration (Billaut et al., 2003). In those studies, as in the present study, the rate of decrease of PP was lower for longer recovery duration. However, repeated sprints with duration similar to this of the present study (20–30 s) were performed with fewer repetitions (2 or 4 repetitions) and longer recovery duration (30 s – 4 min) in those studies (Ainswort et al., 1993; Bogdanis et al., 1996; Dupont et al., 2007; Kohler et al., 2010). In the sole study in which longer repeated sprints (3 × 45 s, workload 53.9 N) with recovery cycling performances of different duration (6, 9 and 12 min, workload 9.8 N) were performed, the power output was significantly less on the 6 min repeated test than on the 9 and 12 min tests, and at least 9 min of recovery cycling was recommended to maintain power output on a repeated 45 s cycling test (Ainswort et al., 1993). As in the study by Ainsworth (1993), the constant peak power values found in the present study for only 3 min recovery duration suggested that longer recovery duration favorably affected the sustainability of power output. However, the studies investigating 20–30 s repeated sprints focused more on recovery types (active and passive) than on recovery duration (Bogdanis et al., 1996; Dupont et al., 2007). Those studies reported that a shorter passive recovery (15 s) protocol between repeated sprints resulted in greater power output values than an active recovery protocol (Dupont et al., 2007). In another study, active recovery with longer duration (4 min) and lower intensity in 2 × 30 s sprints had positive effects on anaerobic performance (Bogdanis et al., 1996). Both studies showed that passive recovery produced positive effects in shorter recovery duration to restore performance, whereas active recovery had a positive effect on anaerobic performance in longer recovery duration. In the present study, 3 min passive recovery affected peak power positively, although the study was not focused on the recovery protocol. In terms of physiological processes of recovery, this finding suggested that 3 min recovery resulted in the replenishment of tissue oxygen stores, PCr resynthesis, lactate metabolism, and the removal of inorganic phosphate (Bogdanis et al., 1995; Faria et al., 2005).

In this study, MP significantly decreased in all recovery duration. Longer recovery periods between shorter repeated sprints had been found to produce a lower rate of decrease in MP values (Glaister et al., 2005; Billaut and Basset, 2007). However, duration of the sprints performed in those studies was 8–10 s, duration that is more likely to affect PP than MP. It had also been shown that longer recovery duration between longer repeated sprints produced a smaller decrease in MP (Ainswort et al., 1993). In the present study, the number and duration of repeated sprints were relatively greater. At this duration and number of repetitions, 1, 2 and 3 min recovery periods were not enough to sustain MP. The intensity of the repetitions in repeated sprints is directly related to the duration of the preceding sprint. In a study in which the power output values obtained from 30 s sprints performed after a 10 s sprint and a 20 s sprint were analyzed, it was found that the PP values for the 10 s sprint were similar to those of the subsequent 30 s sprint, however, the MP values were different. Furthermore, for the 30 s sprint performed after the 20 s sprint, both PP and MP values found for the 30 s sprint were lower than the corresponding values for the preceding 20 s sprint and lower than the corresponding values for a 30 s sprint performed after a 10 s sprint (Bogdanis et al., 1998). The decrease observed in MP in the present study is consistent with the results of previous studies. The reason for all the decreases in MP found for all recovery duration in the present study might be that restoration of MP would require higher rates of ATP regeneration. The incomplete resynthesis of PCr and a possible reduction of glycolysis (35%) owing to elevated H^+^ are expected to reduce anaerobic ATP regeneration (51%) (Bogdanis et al., 1998), while the increase in aerobic metabolism is most likely not significant to compensate (Bogdanis et al., 1996). The FI values were higher only in the 1 min group. This finding suggested that 1 min recovery duration caused more fatigue. On the other hand, the fatigue index was the least reliable of the three Wingate test indices, and its validity was questioned as it largely depends on aerobic performance. Nonetheless, the validity of mean power as an index of anaerobic capacity is as questionable as the validity of the fatigue index (Driss and Vandewalle, 2013).

HR values after repeated WTs were similar for all recovery duration. This result indicated that cyclists generated similar metabolic responses in repeated sprints. However, HR values after recovery intervals of the 1min group were higher than those of the 2 and 3 min groups. The results of the present study showed that HR responses after WTs were similar and submaximal. Chamari et al. (1995) reported a submaximal HR of 83% in an exercise protocol of 6 s sprints with 5 min recovery duration. This submaximal HR response was attributed to the long recovery periods and to the test characteristics, as the test maximally involved the subject’s muscles without overloading the cardioventilatory system (Chamari et al., 1995). Additionally, the same study found that HR response values for the 2^nd^ and 3^rd^ test and recovery were significantly higher than the 1^st^ test and recovery values. The HR responses after recovery reported in that study were similar to those of the present study. In addition, the similar fatigue index levels observed in all recovery duration suggested that the HRs after the WT were also similar.

The present study has a few limitations. The first was the relatively small sample size of highly fit individuals, which most likely decreased our statistical power to detect repeated anaerobic performance differences in trained men. Future studies using a larger sample size are needed to confirm these findings. The second limitation was diet control: the participants were informed about dietary measures, however, diet was not controlled. Thirdly, the current data set is limited to elite athletes, and it might not be generalized to sedentary adults or elderly individuals. The major limitation may be related to the lack of biochemical evaluations, most of all LA and HCO_3_^−^ variables.

## Conclusions

In 4 × 30 s repeated-sprint performances, PP decreased in 1 and 2 min recovery duration, but did not change in the 3 min recovery duration, whereas MP decreased in all recovery periods. However, repeated sprints with 1 min recovery duration resulted in greater fatigue. These results indicate that recovery duration had a greater effect on peak power than on mean power.

## Figures and Tables

**Table 1:** Peak power (PP) output values during repeated Wingate tests with different recovery duration. Values are means (±SD).

Recovery duration	PP (W)						
			
	WT1	WT2	WT3	WT4	D	R	DxR
1 min	731.43±110.04	661.01±91.50	594.23±81.67^a^,^b^	630.44±81.75^a^			
2 min	738.96±116.92	689.23±111.44^a^	633.70±90.18^a^	622.85±97.23^a^	0.32	23.34*	1.43
3 min	716.00±118.26	696.94±107.84	663.61±107.40	655.41±95.85			

* p<0.05

a within-groups significantly different from WT1 (p<0.05)

b p<0.05, within-groups significantly different from WT2 (p<0.05)

**Table 2:** Mean power (MP) output values during repeated Wingate tests with different recovery duration. Values are means (±SD).

Recovery duration	MP (W)						
			
	WT1	WT2	WT3	WT4	D	R	DxR
1 min	590.14±74.68	512.39±51.91^a^	461.41±55.66^a^,^b^	468.01±43.20^a^,^b^			
2 min	591.37±75.51	534.16±62.41^a^	475.45±49.26^a^,^b^	468.00±53.60^a^,^b^	0.81	86.55*	1.72
3 min	585.09±76.11	544.07±64.08^a^	505.71±58.5^a^	503.51±43.74^a^,^b^			

* p<0.05

a within-groups significantly different from WT1 (p<0.05)

b within-groups significantly different from WT2 (p<0.05)

**Table 3:** Fatigue index (FI) values during repeated Wingate tests with different recovery duration. Values are means (±SD).

Recovery duration	FI (%)						
			
	WT1	WT2	WT3	WT4	D	R	DxR
1 min	41.14±6.27	44.20±9.57	45.85±9.4	52.16±10.92^a^			
2 min	45.25±8.94	46.40±8.82	48.71±8.54	48.56±10.75	0.11	8.73*	0.99
3 min	42.54±10.27	44.83±10.88	47.57±9.00	49.15±11.12			

* p<0.05

a within-groups significantly different from WT1, WT2, WT3 (p<0.05)
